# Association Between Potentially Excess Oxygen Exposure and Clinical Outcomes Among Mechanically Ventilated Children in a Tertiary Care Pediatric Intensive Care Unit: A Prospective Observational Study

**DOI:** 10.7759/cureus.110571

**Published:** 2026-06-10

**Authors:** Jamuna Rani, Umesh Pandwar, Navindana, Manjusha Goel

**Affiliations:** 1 Paediatrics, Gandhi Medical College, Bhopal, Bhopal, IND

**Keywords:** cumulative fio₂ exposure, hyperoxia, length of stay, mechanical ventilation, mortality, oxygen toxicity, pediatric intensive care

## Abstract

Background: Oxygen therapy is an essential component of pediatric intensive care; however, liberal administration may contribute to oxidative stress, alveolar injury, and adverse clinical outcomes. Evidence from adult and limited pediatric studies suggests that supraphysiological oxygen exposure may increase mortality and prolong recovery, yet prospective data in mechanically ventilated children remain scarce, particularly from low- and middle-income countries.

Objectives: To determine the association between cumulative fraction of inspired oxygen (FiO₂) exposure and mortality among mechanically ventilated children aged one month to five years and to evaluate its relationship with the duration of hospital stay.

Methods: A prospective observational study was conducted in the Pediatric Intensive Care Unit (PICU) of a tertiary hospital in Bhopal, India, from June 2024 to December 2025. Consecutive children aged one month to five years requiring invasive ventilation for more than 24 hours were enrolled. Cumulative FiO₂ exposure (sum of hours at each FiO₂ level × duration) was calculated from ventilator logs. Demographic, nutritional, and clinical parameters were recorded using a pre-tested proforma. Outcomes were classified as survived or death. Statistical analyses were performed using Stata v14.2; logistic regression identified mortality predictors, and Spearman's correlation assessed the association between FiO₂ exposure and length of hospital stay.

Results: A total of 140 children were included (median (IQR) age 16 (8-36) months; 49.3% male). The median cumulative FiO₂ exposure was 72 (48-84) hours. Mortality was 6.4% (n = 9), and the survival rate was 93.6% (n = 131), comprising 81.4% discharged and 12.1% left against medical advice (LAMA). On multivariable logistic regression, cumulative FiO₂ exposure independently predicted mortality (aOR: 1.22; 95% CI: 1.02-1.46; p = 0.027) after adjustment for age, sex, nutritional status, system involvement, sepsis, and ventilation duration. Male sex (aOR: 2.38; 95% CI: 1.02-5.56; p = 0.046) was also a significant predictor. A strong positive correlation existed between cumulative FiO₂ exposure and length of hospital stay (Spearman r = 0.74; p < 0.001), indicating dose-dependent prolongation of hospitalization.

Conclusion: In this prospective cohort from a tertiary PICU in central India, higher cumulative FiO₂ exposure independently predicted mortality and longer hospital stay. These findings highlight the urgent need for rational oxygen titration and structured pediatric oxygen stewardship to reduce hyperoxia-related harm.

## Introduction

Oxygen therapy remains a cornerstone of pediatric intensive care, particularly among mechanically ventilated children, to correct hypoxemia and maintain tissue perfusion. However, there is growing recognition that liberal oxygen administration may cause harm via oxidative stress, cellular injury, and organ dysfunction [1,2]. A systematic review and meta-analysis of critically ill children found that arterial hyperoxemia was associated with increased mortality (adjusted OR (aOR): ≈1.59; 95% CI: 1.00-2.51) after pooling 11 studies (n ≈ 23,204) [3]. Pre-clinical and adult data show that supraphysiological arterial oxygen tensions (PaO₂) may enhance reactive oxygen species (ROS) formation, lipid peroxidation, endothelial dysfunction, and inflammation pathways that adversely affect pulmonary, cardiac, and neurological systems [4].

In pediatric populations, these concerns may be accentuated due to immature antioxidant defences and different physiological responses to oxygen exposure. Observational data report that in mechanically ventilated children, hyperoxemia (e.g., PaO₂ ≥ 300 mmHg) was independently associated with higher in-hospital mortality (aOR: 1.78; 95% CI: 1.36-2.33) in a large cohort (n ≈ 6,250) of children with arterial blood gas measurements [5]. A recent study of children with severe bronchiolitis requiring invasive ventilation found moderate-to-high-dose pulmonary oxygen exposure and frequent potential oxygen overuse. Although these findings are compelling, significant heterogeneity persists in definitions of hyperoxia (e.g., PaO₂ thresholds, duration of exposure, FiO₂ levels), and the specific context of mechanical ventilation in children remains under-examined [3].

The mechanistic rationale linking excess oxygen to worse outcomes in ventilated children includes alveolar injury, surfactant dysfunction, absorption atelectasis, inflammatory lung injury, and prolongation of ventilator dependence [6,7]. Current oxygenation practices in many Pediatric Intensive Care Units (PICUs) are largely guided by clinical experience rather than robust, evidence-based dose-response data in mechanically ventilated children. Consequently, there remains a significant gap in understanding how cumulative oxygen exposure influences survival and recovery in this vulnerable population. The present study aimed to address this gap by examining the association between cumulative excess oxygen exposure and major clinical outcomes among mechanically ventilated children aged one month to five years. Specifically, it evaluated the relationship between oxygen exposure and mortality as the primary outcome and explored its impact on the duration of hospital stay as a secondary outcome.

## Materials and methods

This prospective observational study was conducted in the PICU of a tertiary care hospital, Bhopal, Madhya Pradesh, India, over an 18-month period from June 2024 to December 2025. The PICU functions as a tertiary-level referral center catering to critically ill pediatric patients requiring advanced respiratory and hemodynamic support.

The study included all children aged one month to five years who required invasive mechanical ventilation for more than 24 hours during the study period. Sample size was calculated using standard single-proportion methods, resulting in a minimum of 138 participants. A consecutive sampling technique was applied until the desired number of participants was achieved. Children with known chronic systemic illnesses, such as congenital heart disease, chronic lung disease, or neuromuscular disorders, and those with recent hospitalization within the preceding 14 days were excluded to avoid confounding by pre-existing conditions.

Data were collected using a pre-tested semi-structured proforma designed by the investigators after a review of published literature and expert consultation. The instrument included socio-demographic data (age and gender), primary diagnosis, and major systemic involvement (respiratory, cardiovascular, hematological, central nervous system, and sepsis status). Nutritional status was classified using weight-for-height or weight-for-length Z-scores as > -2 SD, between -2 and -3 SD, and < -3 SD, as per WHO growth standards [8].

Oxygenation data were captured directly from ventilator monitoring records. The FiO₂ administered at each level (0.3, 0.4, 0.5, 0.6, 0.7, 0.8, and 0.9) and its duration in hours were recorded continuously from the day of intubation until the day of extubation. The cumulative FiO₂ exposure was computed for each patient as the sum of hours spent at each FiO₂ level. Clinical outcomes were classified as (i) survived and (ii) death. The length of hospital stay (in hours) was documented from admission to discharge or death.

Prior to initiation, the data collection tool underwent pre-testing on ten non-study patients to ensure clarity, completeness, and inter-observer agreement. Modifications were made based on pilot feedback. Data were extracted from patient case sheets and ventilator logs, including FiO_2_ readings, timing of intubation/extubation, and outcome data. Training sessions included mock data entry and cross-checking to ensure consistency. Daily verification of ventilator oxygen analyzers and pulse oximeters was carried out by the biomedical technician team to maintain calibration accuracy. Data entry was performed using Microsoft Excel 2019 (Microsoft® Corp., Redmond, WA), and all entries were cross-verified by the senior investigator to prevent transcription errors. Data quality control included weekly reviews of source documents and random cross-checking of 10% of records. De-identified data were stored on a password-protected system accessible only to the investigators.

All analyses were conducted using Stata (version 14.2; StataCorp, College Station, TX). Continuous variables, such as age, FiO₂ duration, and hospital stay, were summarized as mean ± standard deviation (SD) or median with interquartile range (IQR), depending on normality assessed using the Shapiro-Wilk test. Categorical variables, such as sex, systemic involvement, and outcome, were expressed as frequencies and percentages. The association between total FiO₂ exposure (per 10-hour increment) and mortality was assessed using binary logistic regression, with results presented as odds ratios (OR) and 95% confidence intervals (CI). Spearman's correlation and linear regression were used to evaluate the relationship between total FiO₂ exposure and length of hospital stay. A two-sided p-value < 0.05 was considered statistically significant.

Ethical approval was obtained from the Institutional Ethics Committee (Ref. No. 16188/MC/IEC/2024). Written informed consent was obtained from parents or legal guardians prior to inclusion.

## Results

A total of 140 mechanically ventilated children aged one month to five years were included in the analysis. The median (IQR) age was 16 (8-36) months, and 69 (49.3%) were male. Nutritional assessment based on weight-for-height/length Z-score showed that 79 (56.4%) children had normal nutritional status (> -2 SD), 41 (29.3%) had moderate undernutrition (-2 to -3 SD), and 20 (14.3%) had severe undernutrition (< -3 SD). System involvement at admission was most frequently observed in the central nervous system in 85 (60.7%) children, followed by the respiratory system in 65 (46.4%), the hematological system in 20 (14.3%), and the cardiovascular system in 11 (7.9%), while sepsis was present in 31 (22.1%) children (Table 1).

**Table 1:** Baseline Demographic and Clinical Characteristics of the Study Population (n = 140) Data are presented as n (%) for categorical variables and median (interquartile range, IQR) for continuous variables. No statistical comparison was performed for baseline characteristics. A p-value < 0.05 was considered statistically significant. Nutritional status classified per WHO growth standards using weight-for-height/length Z-scores. System involvement categories are not mutually exclusive; one patient may have multiple systems involved. SD = Standard deviation

Characteristic	n (%)/Median (IQR)
Age (months)	16 (8-36)
Gender	
Male	69 (49.3%)
Female	71 (50.7%)
Nutritional Status (Weight-for-Height/Length Z-Score)	
> -2 SD (Normal)	79 (56.4%)
-2 to -3 SD (moderate undernutrition)	41 (29.3%)
< -3 SD (severe undernutrition)	20 (14.3%)
System Involvement at Admission	
Respiratory system	65 (46.4%)
Cardiovascular system	11 (7.9%)
Hematological system	20 (14.3%)
Central nervous system	85 (60.7%)
Sepsis	31 (22.1%)

The most commonly administered FiO₂ level was 0.6, with a median exposure duration of 36 (12-36) hours, followed by FiO₂ of 0.5 and 0.4, each with a median exposure duration of 24 hours. The median cumulative FiO₂ exposure was 72 (48-84) hours. The median duration of mechanical ventilation was 3 (2-3) days, and the median length of hospital stay was 120 (54-240) hours. Of the total cohort, 131 (93.6%) children survived (including 114 (81.4%) discharged and 17 (12.1%) left against medical advice), while nine (6.4%) children died (Table 2).

**Table 2:** Oxygen Exposure Profile and Clinical Course of Mechanically Ventilated Children (n = 140) Data are presented as median (IQR) for continuous variables and n (%) for categorical variables. No statistical comparisons were performed for this table. A p-value < 0.05 was considered statistically significant. FiO₂ = Fraction of inspired oxygen; IQR = Interquartile range; LAMA = Left against medical advice Survived = Discharged + LAMA. LAMA patients were alive at the time of departure and were categorized as survivors for all analyses. Cumulative FiO₂ exposure = sum of hours at each FiO₂ level from intubation to extubation, derived from ventilator logs.

Parameter	Median (IQR)	No. Exposed n (%)
FiO₂ level (hours)	-	-
0.3	24 (12-24)	17 (12.1%)
0.4	24 (21-24)	84 (60.0%)
0.5	24 (12-36)	72 (51.4%)
0.6	36 (12-36)	101 (72.1%)
0.7	24 (24-36)	61 (43.6%)
0.8	24 (12-24)	13 (9.3%)
0.9	26 (24-40)	29 (20.7%)
Cumulative FiO₂ exposure (hours)	72 (48-84)	-
Duration of mechanical ventilation (days)	3 (2-3)	-
Length of hospital stay (hours)	120 (54-240)	-
Clinical outcome	-	-
Survived (discharged + LAMA)	-	131 (93.6%)
Discharged	-	114 (81.4%)
Left against medical advice (LAMA)	-	17 (12.1%)
Death	-	9 (6.4%)

When stratified by tertiles of cumulative FiO₂ exposure, 44 (93.6%), 45 (95.7%), and 42 (91.3%) children survived in the low (T₁), mid (T₂), and high (T₃) exposure groups, respectively, while deaths occurred in three (6.4%), two (4.3%), and four (8.7%) children in the corresponding tertiles (Table 3). The difference in mortality proportions across tertiles was not statistically significant (χ² for trend = 1.24; p = 0.29).

**Table 3:** Clinical Outcomes Across Cumulative FiO₂ Exposure Tertiles (n = 140) Survived = Discharged + Left Against Medical Advice (LAMA) Tertile boundaries were based on the rank distribution of cumulative FiO₂ exposure. Data are presented as n (%). A chi-square test for trend was used to compare mortality proportions across tertiles (χ² = 1.24; p = 0.29). A p-value < 0.05 was considered statistically significant.

FiO₂ Exposure Tertile	Exposure Range (h)	Survived* n (%)	Death n (%)	Total n (%)
T₁ (Low exposure)	≤ 54 h	44 (93.6%)	3 (6.4%)	47 (33.6%)
T₂ (Mid exposure)	55-84 h	45 (95.7%)	2 (4.3%)	47 (33.6%)
T₃ (High exposure)	≥ 85 h	42 (91.3%)	4 (8.7%)	46 (32.8%)
Total	-	131 (93.6%)	9 (6.4%)	140 (100%)

Predictors of mortality were evaluated using binary logistic regression analysis (Table 4). In the unadjusted analysis, cumulative FiO₂ exposure (odds ratio (OR): 1.25; 95% confidence interval (CI): 1.05-1.48; p = 0.012) and male sex (OR: 2.45; 95% CI: 1.04-5.77; p = 0.041) were significantly associated with mortality. After adjustment for age, nutritional status, system involvement, and sepsis, cumulative FiO₂ exposure remained independently associated with mortality (aOR: 1.22; 95% CI: 1.02-1.46; p = 0.027), as did male sex (aOR: 2.38; 95% CI: 1.02-5.56; p = 0.046). Age, nutritional status, system involvement, and sepsis were not significantly associated with mortality in the adjusted model.

**Table 4:** Logistic Regression Analysis for the Predictors of Mortality Among Mechanically Ventilated Children (n = 140) Data are presented as odds ratios (OR) and adjusted odds ratios (aOR) with 95% confidence intervals (CI). Binary logistic regression analysis was used to identify predictors of mortality. A p-value < 0.05 was considered statistically significant. Outcome: Death (n = 9) vs. Survived (n = 131; Discharged + LAMA). All 140 children were included; LAMA cases were merged with discharged patients as survivors. Reference category: OR = 1.00 indicates the baseline group for comparison. Model building: Variables with p < 0.20 in univariate analysis and clinically relevant variables were included in the multivariable model. Cumulative FiO₂ exposure was modeled per 10-hour increment; age was entered as a continuous variable. Statistical analysis was performed using Stata (version 14.2; StataCorp, College Station, TX).

Predictor Variable	Deaths n (%)	Unadjusted OR (95% CI)	p-value	Adjusted aOR (95% CI)	p-value
Cumulative FiO₂ exposure (per 10-h increment)	9 (6.4%)	1.25 (1.05-1.48)	0.012	1.22 (1.02-1.46)	0.027
Age (months, continuous)	-	0.98 (0.95-1.02)	0.31	0.99 (0.95-1.03)	0.54
Gender					
Female (Reference)	3 (4.2%)	1.00	-	1.00	-
Male	6 (8.7%)	2.45 (1.04-5.77)	0.041	2.38 (1.02-5.56)	0.046
Nutritional Status (Z-score)					
> -2 SD (Normal, Reference)	4 (5.1%)	1.00	-	1.00	-
-2 to -3 SD (Moderate undernutrition)	3 (7.3%)	1.47 (0.32-6.64)	0.62	1.32 (0.27-6.46)	0.72
< -3 SD (Severe undernutrition)	2 (10.0%)	2.05 (0.35-11.9)	0.42	1.88 (0.29-12.0)	0.48
System Involvement					
No (Reference)	3 (4.1%)	1.00	-	1.00	-
Yes	6 (8.8%)	2.24 (0.58-8.59)	0.24	1.98 (0.49-8.00)	0.32
Sepsis					
No (Reference)	6 (5.5%)	1.00	-	1.00	-
Yes	3 (9.7%)	1.82 (0.42-7.87)	0.42	1.61 (0.33-7.76)	0.55

**Figure 1:**
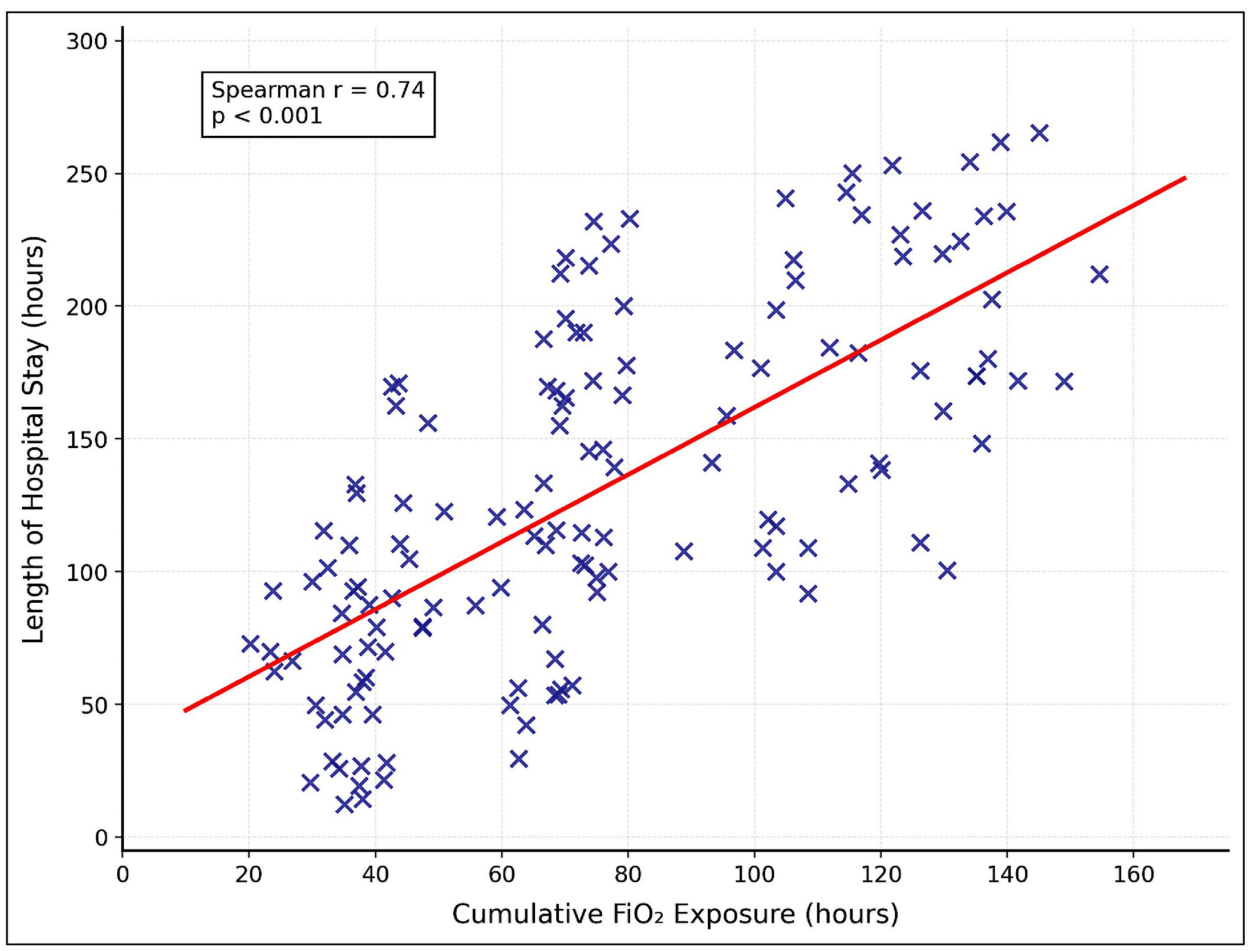
Correlation Between Cumulative FiO₂ Exposure (Hours) and Length of Hospital Stay (Hours) Among Mechanically Ventilated Children (n = 140) Data are presented as individual observations, with each cross (×) representing one patient. The red line represents the fitted linear regression line. Correlation was assessed using Spearman’s rank correlation coefficient (r = 0.74; p < 0.001). A p-value < 0.05 was considered statistically significant. Note: The y-axis represents the length of hospital stay in hours (corrected from the original figure, which was labeled as days). FiO₂ = Fraction of inspired oxygen; IQR = Interquartile range; PICU = Pediatric Intensive Care Unit

A correlation analysis demonstrated a statistically significant association between cumulative FiO₂ exposure and length of hospital stay. Spearman's rank correlation coefficient indicated a strong positive correlation (r = 0.74; p < 0.001), indicating a strong positive correlation between cumulative FiO₂ exposure and length of hospital stay (Figure 1).

## Discussion

Our study found that higher cumulative exposure to supplemental oxygen in mechanically ventilated children was significantly associated with worse clinical outcomes. Specifically, each 10-hour increase in FiO₂ exposure independently increased the odds of mortality by 22%, even after adjusting for relevant clinical variables. Additionally, there was a strong positive correlation between total oxygen exposure and the length of hospital stay, suggesting that a greater oxygen burden may contribute not only to increased risk of death but also to prolonged recovery in this vulnerable population. Across rank-based tertiles of cumulative FiO₂ exposure, mortality showed a modest but non-significant increase (6.4% in T₁, 4.3% in T₂, and 8.7% in T₃; χ² for trend = 0.29). This pattern is consistent with a possible dose-response relationship between higher cumulative oxygen exposure and adverse outcomes, although statistical significance was not achieved, likely due to the small number of deaths (n = 9).

Comparison with existing literature

Our results are closely aligned with the study by Geva et al. [9], which validated and extended the concept of cumulative excess oxygen exposure (CEOE) in pediatric intensive care. In their large retrospective cohort study (n = 3,354), patients in the highest CEOE quartile experienced an 87% increased risk of mortality compared with those in the lowest quartile. Their operational definition of CEOE - mean hourly FiO₂ above 0.21 when SpO₂ was ≥ 95% - focuses on potentially avoidable hyperoxia and provides a physiologically relevant framework for evaluating oxygen burden. Although our study used total cumulative FiO₂ duration rather than adjusting for SpO₂, the consistent dose-response relationship observed in both studies highlights the clinical importance of minimizing unnecessary oxygen administration.

Similar conclusions were drawn by Balcarcel et al. [10], who demonstrated that excessive oxygen supplementation during the first 24 hours of mechanical ventilation was independently associated with both increased in-hospital mortality and the development of multiple organ dysfunction syndrome (MODS) by day seven. Their study's large sample size (n = 5,406), robust multivariate analysis, and focus on early oxygen burden lend strong external validity. The alignment of their findings with ours supports a growing consensus that excess oxygen, even when SpO₂ is within a "normal" range, can trigger harmful pathophysiological cascades - including oxidative stress, mitochondrial injury, endothelial dysfunction, and inflammation - mechanisms that are well-established in preclinical models and are especially relevant in children due to immature antioxidant defenses [11].

In contrast, a study by Naz et al. [12] conducted in a tertiary PICU in Pakistan reported no significant association between CEOE and mortality or MODS. The divergence in findings may be explained by several methodological and contextual factors. Their study had a smaller sample size (n = 191), and the use of SpO₂ rather than PaO₂ as a surrogate for oxygenation may have introduced measurement bias. Furthermore, the exposure window was limited to the first 24 hours of ventilation, potentially missing the cumulative effects of prolonged oxygen therapy. The inclusion of both mechanically ventilated and high-flow nasal cannula (HFNC)-supported patients may have also introduced heterogeneity, making their findings less comparable to ours, which focused exclusively on invasive ventilation.

The observed Spearman correlation (r = 0.74, p < 0.001) denotes a strong positive association, suggesting that higher cumulative oxygen exposure is consistently linked with longer hospital stay durations. This strength of correlation highlights the potential dose-dependent impact of oxygen burden on recovery time in ventilated children.

Mechanistically, the adverse effects of hyperoxia are supported by biological plausibility and experimental studies. Prolonged exposure to high FiO₂ increases the production of ROS, leading to alveolar epithelial damage, surfactant dysfunction, and inflammatory lung injury [13,14]. Rachmale et al. [15] demonstrated that adult ICU patients exposed to excessive FiO₂ (> 0.5 despite SpO₂ > 92%) had significantly worse oxygenation indices at 48 hours, longer ventilation duration, and extended ICU stays. Although this study was conducted in adults, the underlying mechanisms are likely to be amplified in pediatric patients, whose lung parenchyma and antioxidant systems are still developing.

Clinical implications

The findings of this study carry significant clinical implications for pediatric intensive care practice. Our results underscore the need for more cautious and individualized oxygen titration strategies in mechanically ventilated children, challenging the historically liberal approach to oxygen therapy. Given the clear association between higher cumulative FiO₂ exposure and both increased mortality and prolonged hospitalization, oxygen should be treated not merely as a supportive intervention but as a pharmacologic agent with potential for harm when administered excessively. These results advocate for the development of standardized oxygen stewardship protocols in PICUs, including defined upper FiO₂ limits, real-time FiO₂-SpO₂ monitoring systems, and training programs for frontline staff. Incorporating oxygen exposure metrics into early-warning systems or ventilator dashboards may allow for proactive adjustments, minimizing unnecessary hyperoxia. Importantly, these strategies should be adapted to resource-constrained settings, where advanced monitoring tools may be limited but where the risk-benefit balance of oxygen therapy may be even more critical [16,17].

Strengths and limitations

This study has several notable strengths. It is among the few prospective investigations to evaluate cumulative oxygen exposure and its association with clinical outcomes in a low- and middle-income country setting. The use of continuous FiO₂ documentation directly from ventilator logs enhances the accuracy of exposure assessment, and the analysis was adjusted for multiple potential confounders, including nutritional status and system involvement. The study also benefited from clearly defined inclusion and exclusion criteria and a focused pediatric age group, enhancing internal validity.

However, certain limitations must be acknowledged. First, the study was conducted at a single tertiary care center, which may limit the generalizability of findings. Second, SpO₂ or PaO₂ values were not incorporated into the exposure definition, which precluded calculation of CEOE or oxygenation indices commonly used in other studies. Third, long-term outcomes, such as neurodevelopmental sequelae, post-discharge morbidity, or ventilator-associated complications, were not assessed. Finally, the observational design limits causal inference, though the strength and consistency of the associations support the plausibility of the observed effects.

## Conclusions

In this prospective study of mechanically ventilated children, higher cumulative FiO₂ exposure was associated with increased mortality and prolonged hospital stay. However, because PaO₂ and other measures of oxygenation were not incorporated into the analysis, and residual confounding by illness severity cannot be excluded, these findings should be interpreted as an association rather than evidence of a causal effect. It is possible that sicker children received higher FiO₂ as part of appropriate clinical management. Further studies are needed to determine whether oxygen exposure independently influences outcomes in critically ill children.
